# Persistent median artery and communicating branch related to the superficial palmar arch

**DOI:** 10.1038/s41598-023-50935-2

**Published:** 2024-01-02

**Authors:** Marko Simić, Marko Bumbaširević, Darko Jović, Nikola Bogosavljević, Marko Vujačić, Dražan Erić, Jelena Boljanović, Milan Milisavljević, Aleksandra Dožić, Mila Ćetković

**Affiliations:** 1 Clinic for Orthopedic Surgery and Traumatology, University Clinical Center of Serbia, Faculty of Medicine, University of Belgrade, Belgrade, Serbia; 2 Clinic of Plastic and Reconstructive Surgery, University Clinical Centre of Republic of Srpska, Banja Luka, Republic of Srpska Bosnia and Herzegovina; 3 Institute for Orthopedic Surgery “Banjica”, Faculty of Medicine, University of Belgrade, Belgrade, Serbia; 4 Department of Plastic and Reconstructive Surgery, Al Emadi Hospital, Doha, Qatar; 5 Laboratory for Vascular Morphology, Institute of Anatomy, Faculty of Medicine, University of Belgrade, Belgrade, Serbia; 6 Academy of Medical Sciences, Serbian Medical Association, Belgrade, Serbia; 7 Institute of Anatomy, Faculty of Dental Medicine, University of Belgrade, Belgrade, Serbia; 8 Institute of Histology and Embryology, Faculty of Medicine, University of Belgrade, Belgrade, Serbia

**Keywords:** Anatomy, Medical research

## Abstract

Microvascular surgery, plastic and reconstructive hand surgery, and coronary artery bypass surgery call for a microanatomical study of the branching pattern of the superficial palmar arch (SPA). For the anatomical analysis, we used a group of 20 dissected human hands injected with 4% formaldehyde solution and a 10% mixture of melted gelatin and India ink. The morphometric study was performed on 40 human hands of adult persons injected with methyl-methacrylate fluid into the ulnar and radial arteries simultaneously and afterwards corroded in 40% KOH solution for the preparation of corrosion cast specimens. The mean diameter of the SPA, between the second and third common palmar digital arteries, was 1.86 ± 0.08 mm. We identified the persistent median artery (PMA) in 5% of hands. We distinguished the three main groups of the SPAs according to variations in morphology and branching of the arch: Type 1, the long SPA; Type 2, the middle length SPA; and Type 3, the short SPA found in 27.5% of specimens. The communicating branch (CB), a vessel interconnecting the SPA to the closest branch of the radial artery, is classified into two different morphological groups. The third type of incomplete short arterial arch is the most important of the three groups of SPAs. That short SPA is potentially inadequate for restoring circulation after occlusion or radial artery harvesting for coronary artery bypass.

## Introduction

The superficial palmar arch (SPA), according to the descriptions in different anatomy textbooks, is the final palmar segment of the ulnar artery that is completed with the superficial palmar branch of the radial artery (SPB)^1,2^. It enters the palm lateral to the pisiform bone, medially to the hamate bone, superficial to the flexor retinaculum, and arches laterally, forming the terminal part that is concave proximally^2,3^. It is attached to the deep side of the palmar aponeurosis and lies more superficially than the nerves of the palm. It is commonly described that the three common palmar digital arteries (CPDAs), originating from the superficial palmar arch, extend toward the fingers where they branch into two smaller, proper arteries for two adjacent fingers. The SPA is superficial, easy approachable for dissection, and considered to have a high incidence of variations^1–3^.

The normal embryologic development of the arm arteries begins with the initial vascular plexuses growing by angiogenesis throughout the upper limb bud. During the next period, the new axial axillary arterial trunk enters the extremity bud, remodeling the capillary network. Finally, it forms a central, interosseous artery of the forearm. The median artery is the next developing vessel in early embryonic life, dividing from the previous one, following the median nerve, and terminating as the digital arteries. Finally, after the regression of the median artery, in the 8th week of gestation, the ulnar artery and, with delay, the radial artery, both coming later from the brachial artery, become the dominant supply of the hand, influencing the differentiation of the skeletal parts^4^. If the palmar median artery persists in adults, this embryological remnant becomes perceived as a persistent median artery (PMA), participating in the supply of the median nerve and the formation of the SPA^5^.

The dissection of formalin-fixed hands during the educational courses for medical students, mostly without any kind of injected intra-arterial mixture, was the preferred method for the investigations of the vascular anatomy of the hand. The problems with dissecting small, fragile and deeper positioned vessels contributed to the quality of available presentations, with very little documented precise information on the superficial neurovascular layer of the hand, branching pattern of the SPA, connection with the SPB, and the persistent median artery, with related diameters^6–10^. We introduced the improved anatomical technique using the corrosion vascular casts of the previously injected radial and ulnar arteries for the analyses of exact spatial relations between the arteries of the hand. We presented more accurate data explaining the palmar arterial network, and we performed computer-assisted measurements of intraluminal diameters of the branches^11,12^.

Since the hand is the most mobile part of the body and because any work is practically unthinkable without its participation, it is very often exposed to injury. The topographical relationships and variations of the SPA, diameters and anastomoses are important in vascular and microvascular surgery of the hand. The procedures of vascular posttraumatic repair with anastomosis of small blood vessels under the surgical microscope should be successful and safe for patients^13^. Understanding the hand’s vascular anatomy is important in plastic and reconstructive hand surgery. The palmar circulation with the possibility of variations of the SPA and communication between the branches offers the anatomical basis and allows improved mobilization of a variety of flaps^14^. The radial artery harvesting for the coronary artery bypass graft procedure requires the evaluation of the collateral circulation from the ulnar artery to avoid hand ischemia^15^.

The purpose of our research was to analyze descriptions and data from the published literature on arterial variations of the SPA and to compare them with our precise and simplified vascular patterns. The objectives of this study were to determine the position, branching, anastomoses and topographic relationships and diameters of the terminal segment of the ulnar artery and its communicating branch with implications in orthopedic, microvascular and reconstructive surgery.

## Materials and methods

This microanatomical study of the superficial palmar arch was conducted on two sets of human specimens. The first group contained 20 human hands injected with 4% formaldehyde solution and a 10% mixture of melted gelatin and India ink simultaneously into the ulnar and radial arteries. After one month of fixation, the forearms and hands were meticulously dissected. This group of traditionally prepared and dissected hands showed the main palmar arteries in relationships with the muscles and neighboring nerves.

We used another large group of 40 human hands (19 right, 21 left) of adults (15 female and 25 male), with an average age of 51.2 (from 26 to 65) years, for the preparation of corrosion vascular casts. The solution of methyl methacrylate, mixed shortly before injection with added hardener and color pigment, was injected at the same time into the ulnar and radial arteries. After four hours of polymerization and hardening, we used a 40% solution of potassium hydroxide necessary for corrosion of the soft tissue for the next two weeks. For the final cleaning of digested specimens, we immersed them in hot running water. The corrosion cast specimens of hand arteries were analyzed under a zoom microscope (Leica MZ6) and photographed by a digital photo camera (Leica DFC295). With the use of specific software (Leica Interactive Measurements), we realized different kinds of measurements. Vascular networks of the distal segment of the ulnar artery, anatomical variations, branching mode, collateral and terminal branches and all anastomoses were recorded. The obtained data were introduced into the schematic drawings of every specimen. The research method using the corrosion casts in analyses provided a precise view into the spatial relations between the arteries, offering a much more realistic arterial network than traditional dissections with the permanent possibility of damaging and removing small vessels. This method of preparation and examination of the arteries is more reliable than any other method of research used in morphological studies in this area.

All statistical analyses were performed with the aid of the SPSS 17.0 statistical program (SPSS, Inc., Chicago, IL, USA). The statistical analyses comprised the descriptive statistics (mean values and standard deviations, and minimum and maximum values) of the measured data. This microanatomical study of the superficial palmar arch was conducted on two sets of human specimens from the collection of the Laboratory for Vascular Morphology, Faculty of Medicine Institute of Anatomy. The individuals whose tissue parts were used for the study had signed Informed consent forms prior to their deaths for the use of their bodies for scientific and educational purposes. This study was performed in line with the principles of the Declaration of Helsinki. The study protocol was approved by the Ethics Committee of the Faculty of Medicine, University of Belgrade, Belgrade, Serbia (No. 29/VI-1; Date 19-6-2013). The obtained set of specimens served as a useful demonstration tool in teaching medical students, as an adjunct in the collection of our Museum of Human Anatomy as well as in preparation of the Atlas of the Human Body^12^.

## Results

**Figure 1:**
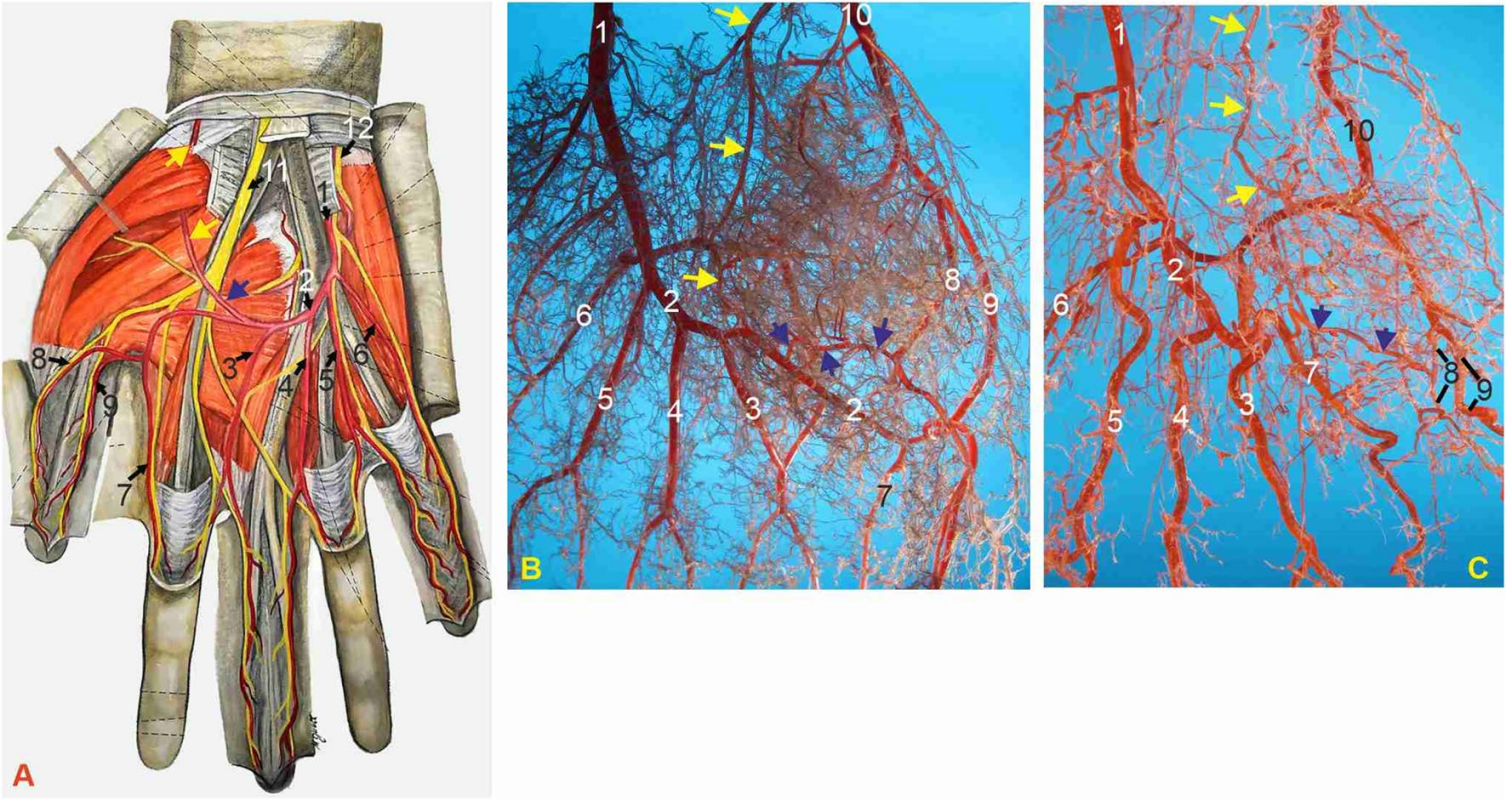
Palmar view of hands with arteries of the superficial neurovascular layer, type 1. (**A**) Drawing of the typically described final anastomosis of the arching terminal part of the ulnar artery (blue arrow) with the palmar branch of the radial artery (yellow arrows) and the widest field of supply including the thumb (modified from Radojević and Bošković^16^). (**B**,**C**) Corrosion casts of left hand palmar arteries: (**B**) subtype a.; (**C**) subtype b. 1—ulnar artery; 2—superficial palmar arch (SPA); blue arrows—communicating branch (CB); yellow arrows—superficial palmar branch of the radial artery (SPB); 3—first CPDA; 4—second CPDA; 5—third CPDA; 6—ulnaris digiti minimi palmar artery; 7—radialis indicis artery; 8—radialis pollicis palmar artery; 9—ulnaris pollicis palmar artery; 10—radial artery; 11—median nerve; 12—ulnar nerve.

**Figure 2:**
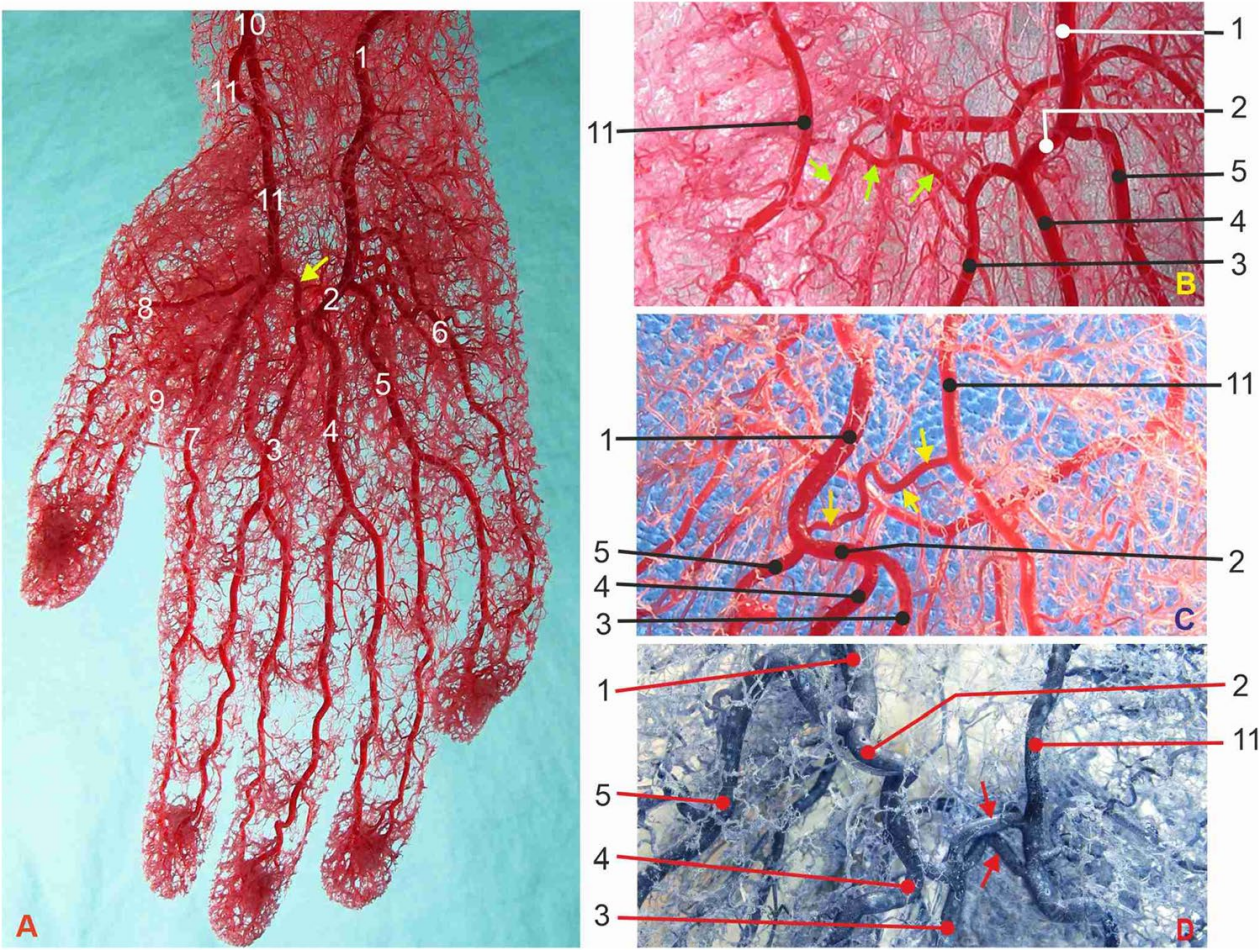
Corrosion casts of four hands with the medium length SPA, type 2, giving off three CPDAs. (**A**) and (**B**) right hand palmar arteries. (**C**) and (**D**) left hand palmar arteries. 1—ulnar artery; 2—superficial palmar arch (SPA); yellow arrows—communicating branch (CB); red arrows—doubled CB; 3—first CPDA; 4—second CPDA; 5—third CPDA; 6—ulnaris digiti minimi palmar artery; 7—radialis indicis artery; 8—radialis pollicis palmar artery; 9—ulnaris pollicis palmar artery; 10—radial artery; 11—prominent superficial palmar branch of the radial artery (SPB).

**Figure 3:**
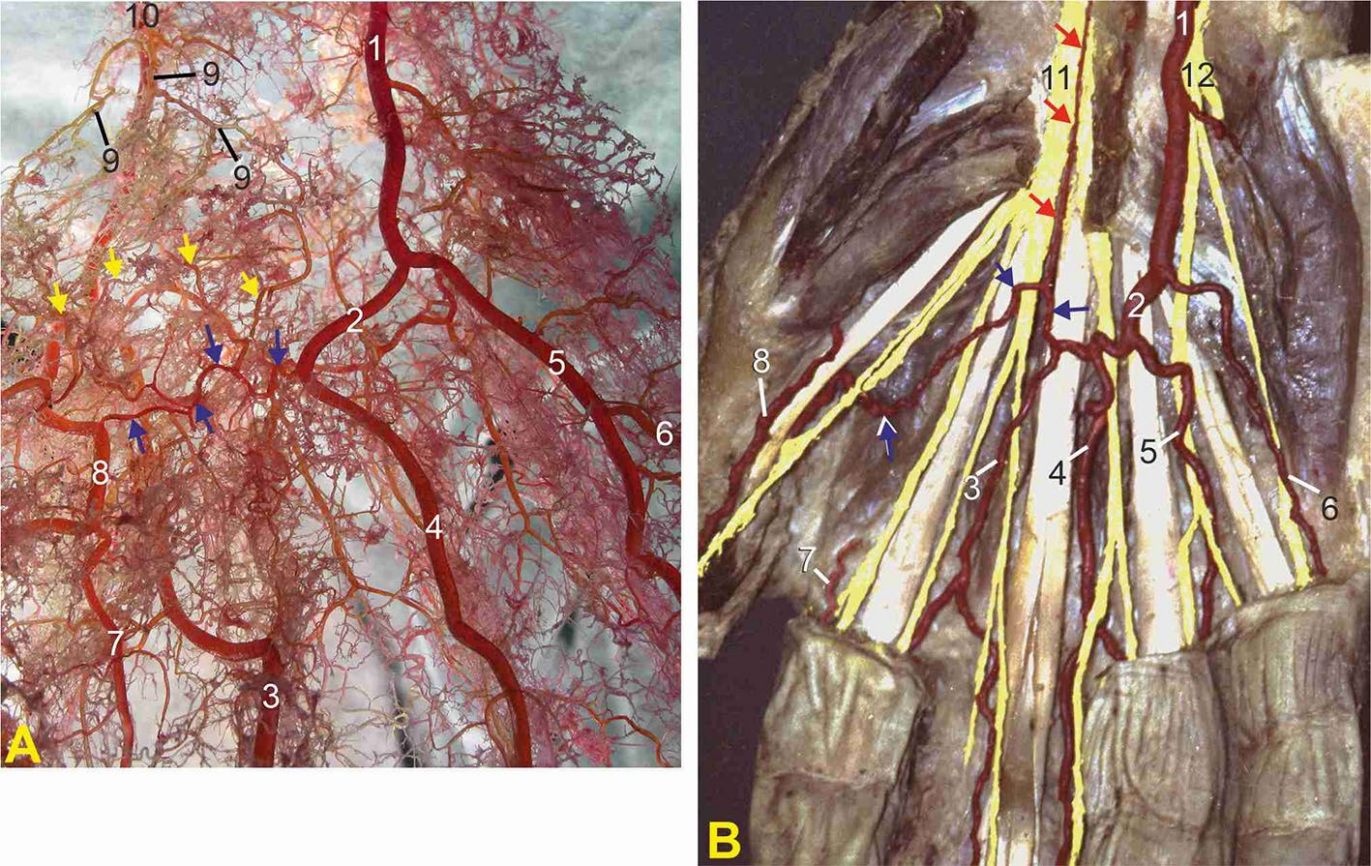
(**A**) Corrosion casts of type 3, the short SPA giving origin to two CPDAs. 1—ulnar artery; 2—superficial palmar arch (SPA); blue arrows—communicating branch (CB); 3—first CPDA; 4—second CPDA; 5—third CPDA; 6—ulnaris digiti minimi palmar artery; 7—radialis indicis artery; 8—radialis pollicis palmar artery; 9—superficial palmar branch of the radial artery (SPB); yellow arrows—anastomotic connections between the CB and SPB; 10—radial artery; 11—median nerve; 12—ulnar nerve. (**B**) Superficial dissection of the right hand showing the persistent median artery (red arrows) joining the CB (blue arrows).

The superficial palmar arch (SPA) is considered a continuation of the ulnar artery (UA) from the point of the beginning of the deep palmar branch (DPB) from the UA. The SPA existed in all 40 studied cases (100%). DPB contributes to the deep neurovascular layer of the hand by completing the deep palmar arch with the terminal part of the radial artery (RA). The UA caliber ranged from 2.5 to 2.75 mm, mean 2.65 ± 0.08 mm, measured immediately proximal to the origin of the DPB. Its diameter gradually decreased as the common palmar digital arteries (CPDAs) separated. The CPDAs terminated by dividing into two proper palmar digital arteries. The measured diameters of the SPA, between the 2nd and 3rd common palmar arteries of the fingers (part of the vessel that always existed), had values from 1.72 to 2.1 mm, on average 1.86 ± 0.08 mm. The average diameter of the CPDAs was 1.72 ± 0.08 mm, with a range of 1.60–1.85 mm (Figs. 1, 2, 3, Table 1).

**Table 1:** Morphometric characteristics of the UA, SPA, CB and CPDAs.

Artery	Frequency, hands (%)	Diameter (mm); range (mean ± SD)
UA	40 (100)	2.5–2.75 (2.65 ± 0.08)
SPA	40 (100)	1.72–2.1 (1.86 ± 0.08)
(1) Long	15 (37.5)	
(2) Medium length	14 (35)	
(3) Short	11 (27.5)	
CB	40 (100)	
Class I	29 (72.5)	0.40–1.0 (0.6 ± 0.21)
Class II	11 (27.5)	0.25–0.38 (0.29 ± 0.04)
CPDAs	40 (100)	1.60–1.85 (1.72 ± 0.08)

*UA* ulnar artery, *SPA* superficial palmar arch, *CB* communicating branch, *CPDA* common palmar digital artery.

We distinguished the three main groups of SPAs according to variations in morphology and branching of the arch: long, medium length, and short SPA.

### Complete SPA; long SPA (Type 1)

*Type 1* A long SPA existed in 15 (37.5%) hands. This was the most complex variant occupying the superficial neurovascular layer of the palm (Fig. 1). Mostly, the SPA of subtype A, after sending the three common palmar digital arteries (CPDAs) and radialis indicis artery (RIA), except in one case of RIA coming from the deep palmar arch, ended by entering into the ulnaris or radialis pollicis palmar artery in 7 out of 11 hands (Fig. 1A,B). In the other 4 cases, subtype B, the SPA terminated as the radialis indicis artery (RIA), with no additional branch to the thumb (Fig. 1C, Table 1).

### Complete SPA; medium length SPA (Type 2)

*Type 2* Medium-length SPA was present in 14 (35%) casts. This arterial pattern is the most commonly described in the literature, giving off the three CPDAs. The first CPDA descends to split into two proper digital arteries for the medial surface of the index finger and the lateral surface of the middle finger (Fig. 2, Table 1).

### Incomplete SPA; short SPA (Type 3)

*Type 3* The short SPA was found in 11 (27.5%) specimens, providing origin for only two CPDAs, the second and the third (Fig. 3A, Table 1).

### Persistent median artery (PMA)

A persistent median artery (PMA) was observed in 1 (5%) out of 20 dissected hands. It passed through the carpal tunnel and vascularized median nerve and participated in the formation of the SPA and the arterial supply of the hand (Fig. 3B).

### Communicating branch (CB)

Communicating branch (CB), the term that we introduced for a vessel interconnecting the SPA to the closest artery, is found in all hands (100%). We described the CB as a separate entity because of its inserted position showing the same calibers at both of its connecting ends, which is usually significantly smaller than the diameter of the interconnected vessels. Regarding the three types of SPA, the CB showed a different appearance. We classified the studied CBs into two different morphological groups: Class I, present in types 1 and 2 of the SPAs, and Class II, characteristic of type 3 SPAs. In the first type (37.5%), when the SPA was long, the CB extended to enter into the ulnaris or radialis pollicis palmar artery (Fig. 1B,C). In a second group of cases (35%) with the typical medium length SPA and prominent SPB, the CB transversely interconnected them by one vessel (Fig. 2A–C), except in one hand having two CBs (Fig. 2D). The diameter of the Class I CBs ranged from 0.40 to 1.00 mm, averaging 0.6 ± 0.21 mm. Finally, the third type of arching vessel, when a short SPA existed (27.5%), was characterized by the longest CB but with the smallest diameter, belonging to Class II of the studied CBs. The slender CBs of these specimens, with an average caliber of 0.29 ± 0.04 mm, from 0.25 to 0.38 mm, connected the short SPA with one of the arteries of the thumb but also had anastomotic branches for the connection with small arteries coming from the small SPBs (Fig. 3A; Table 1).

## Discussion

In the present study, we identified the prevalence of persistent median artery (PMA) in 5% of injected and dissected hands, following the median nerve thorough the carpal canal and entering the distal part of the SPA. Other reports described a more or less similar presence of PMA ranging from 0.9 to 6.6%^17–20^, or from 8.0 to 15.5%^6,7,9^, or a much higher incidence of persistence, varying between 21.0 and 59.7% of hands^8,21,22^ (Table 2).

**Table 2:** Authors of the dissection studies and incidence of the median artery reported in manuscripts.

Authors (year)	Incidence (%)
Ikeda et al. (1988)	0.9
Gellman et al. (2001)	15.5
Fazan et al. (2004)	11.0
Loukas et al. (2005)	21.0
Bilge et al. (2006)	8.0
Natsis te al. (2009)	2.78
Eid et al. (2011)	4.0
Singla et al. (2012)	6.6
Cheruiyot et al. (2017)	59.7
Lucas et al. (2020)	33.3

The presence of the PMA in the region of a carpal tunnel could participate in mechanical compression of the median nerve, resulting in an insufficient blood supply and the appearance of carpal tunnel signs and symptoms. Because of the well-packed elements of the carpal canal content, pressed between the carpal bones and the transverse carpal ligament, a PMA of a larger caliber, aneurysm, atherosclerosis, calcification, or thrombosis of the PMA can be associated with median nerve compression^20,23–25^. Orthopedic surgeons should respect this anatomical variation that could meet during carpal tunnel decompression. In the presented case, we observed the PMA, which participated in the formation of an SPA and ended in a distal part of the SPA, forming a radial-median-ulnar contribution to the arch. The contribution of the PMA to the blood flow of the hand depends on its size and termination. The median-ulnar types of the SPA have also been described, as well as the median-radial pattern and an independent termination of the PMA giving of the 1st and 2nd CPDAs^8,18,26^.

Normal embryonal development explains that arterial variations in the hand are formed by modifications of the primary capillary pattern by means of progression and differentiation of one group or regression of another group of capillaries depending on the hemodynamic predominance of the feeding arteries of the hand: interosseous, median, ulnar and radial. The interarterial connections have a function of maintaining a constant hand supply^4,27^.

The important question in different published articles is the termination of the SPA or the question of a complete or incomplete SPA. The results are presented in numerous classifications made by different authors. The traditional definition of a complete SPA, or radioulnar arch, is related to the existence of an anastomosis between the terminal arching part of the UA and the SPB of the RA^7–10,17,28^. A traditional complete SPA was present in 27.4% of specimens reported by Feigl et al.^28^, 34% by Bilge et al.^9^, 35.5% by Gellman et al.^6^, 40% by Loukas et al.^8^, 44% by Singh et al.^10^, 48.5% by Fazan et al.^7^, and 55.9% by Ikeda et al.^17^. We found this type of complete arch in 35% of cases, and our results are in accordance with the previously published findings of the mentioned authors. The described anastomosis was, unlike the numerous anatomical drawings, never in the form of termination of the SPB into the SPA or end-to-end anastomosis. The second type of medium-length SPA, type 2 in our study, showed an inserted anastomotic vessel between the convexity of the end point of the SPA and the beginning of the first CPDA, or more proximal from the SPA itself in some cases, on one side, and the surface of the SPB of the RA. Because of the specific inserted position, interconnecting two arteries, we named that anastomotic vessel the communicating branch (CB). The CB had the same intraluminal diameters at both ends, with an average value of 0.6 mm. Our original descriptions of the CB and interconnected vessels were realized using vascular arterial casts for this morphological analysis. In all hands with this form of the SPAs and CBs, the SPBs of RAs were always well developed and extended distally in a hand to supply the lateral surface of the index finger and surfaces of the thumb, as described in a previous study^11^. This kind of balanced supply in the superficial arterial palmar plane, with the presence of CB, is an example of equal dominance of the RA and UA.

The second group of complete SPA was characterized by an anastomosis connecting the terminal arching part of the long SPA, type 1 in our study, and the ulnar or radial palmar artery of the thumb, originating from the RA. This type of variant was reported as the ulnar type, exposing the largest field of supply of the SPA from the UA, but the connection with the RA by the CB always existed, as we noticed. Dominant UA sent branches for the supply of four fingers, with a contribution to the thumb vasculature, leaving the thenar area as a field of supply of short SPB^11,29^. This variation was observed in 14% of hands by Bilge et al.^9^, 20.9% by Feigl^28^, 25.5% by Ikeda et al.^17^, 31% by Gellman et al.^6^, 33% by Fazan et al.^7^, 35% by Loukas et al.^8^, and 46% by Singh et al.^10^. We identified complete and long SPA in 37.5% of hands, and our results are similar to previously reported studies by cited authors. Our study showed that in this group of cases with a dominant UA in a superficial arterial palmar plane, the SPA is also anastomosed by the CB, and in 15% of hands, it is additionally anastomosed by the end of the SPA itself, with branches of the RA for the supply of thumb.

The hemodynamic balance between two feeding arteries, ulnar and radial, of the superficial and deep layers of the hand creates the shape of the always present SPB of the RA. Our detailed analysis proved the existence of two morphological types of the SPB: hypoplastic and prominent. The hypoplastic SPB, found in 26 (65%) hands, with an average diameter of 1.18 mm, ranging from 0.8 to 1.5 mm, typically supplied the muscles and skin of the thenar eminence. The fine ramification of the hypoplastic SPB was reach and tree like, distally sending multiple slender anastomotic branches to delicate CB of the short SPA (type 3) of our classification. The same hypoplastic SPB branching pattern existed in the cases with long SPA (type 1), and similar anastomotic small vessels went for the connection with long CB. The prominent SPB, found in 14 (35%) specimens, had an average diameter of 2.0 mm, ranging from 1.7 to 2.75 mm. It was present in cases with the medium length SPA (type 2). The prominent SPB is longer vessel leaving the thenar area to enter into the distal palm for the supply of lateral part of index finger and part of the thumb. The observed relationship existed only in hands with prominent SPB, and the CB had a larger diameter interconnecting the side of the SPB with the SPA. It would be questionable to claim what would be the blood direction within the CB, but the balanced supply of the hand structures from two main parent arteries, ulnar and radial, should be constant and sufficient. We never noticed the existence of straight end-to-end termination of the SPB into the SPA, except in traditional anatomical descriptions and drowings^1,2,16,30^. These findings are in accordance with the results of our previous study^11,31^.

The radial artery is used by surgeons to create a coronary artery bypass. The removal of RA could cause circulatory changes, with a decrease in blood flow through the hand and fingers and postoperative ischemia^15,30^. We proved that in 27.5% of hands, the third type of short SPA of the UA had the smallest field of supply and delicate CB with slender anastomoses with the hypoplastic SPB of the RA. Inadequate collateral circulation after radial artery harvesting could not transmit a compensatory increase in blood flow from the UA for the vascularization of all fingers^32^. The limitation of our study could be the missing study of a deeper neurovascular region of the hand. This analysis of the superficial palmar vascular pattern requires additional research on the deep palmar vascular layer to obtain a precise and complete picture of the collateral circulation via the deep palmar arch.

## Conclusion

The result of our study is a detailed analysis of the superficial palmar vascular pattern of the hand classified into three types. The 1st type was represented by the complete, long SPAs for the supply of four fingers and partially the thumb. The 2nd type included the complete, middle length SPAs supplying the medial half of the index finger and the other three fingers. The 3rd type contained incomplete, short SPAs for the supply of the medial half of the middle finger and the 4th and 5th fingers. In all three groups, the CB, the anastomotic connection between the SPA and a branch of RA, was always present. We highlighted the 3rd type of incomplete, short arterial arch with the smallest field of supply and slender anastomotic connections. Based on the obtained anatomical data, the short described variation of SPAs found in 27.5% of the hands is potentially of surgical and clinical importance after occlusion of the RA or radial artery harvesting for coronary artery bypass. This study showed that knowledge of vascular variations and competence of the SPA before removal of the radial artery is crucial to avoid potential vascular complications in supply to the thumb and the index finger.

## Data Availability

All relevant data are included in the manuscript and its table.

## Abbreviations

CB: Communicating branch
CPDA: Common palmar digital artery
PMA: Persistent median artery
RA: Radial artery
RIA: Radialis indicis artery
SPA: Superficial palmar arch
SPB: Superficial palmar branch
UA: Ulnar artery
